# Genetic and Environmental Contributions To Gender Diversity: A Systematic Review of the Twin Literature

**DOI:** 10.1007/s10519-025-10231-3

**Published:** 2025-09-12

**Authors:** Will Conabere, Louise Bourchier, Sue Malta, Anja Ravine, Ken C. Pang

**Affiliations:** 1 Department of Paediatrics, University of Melbourne, Melbourne, Victoria Australia; 2 Murdoch Children’s Research Institute, Parkville, VIC Australia; 3 Sexual Health Unit, University of Melbourne, Melbourne, Victoria Australia; 4 Twins Research Australia, University of Melbourne, Melbourne, Victoria Australia; 5 Department of Adolescent Medicine, The Royal Children’s Hospital Melbourne, Parkville, VIC Australia

**Keywords:** Transgender, Gender identity, Twin studies, Heritability, Prenatal hormone transfer, Genetics, Concordance

## Abstract

Amidst growing visibility of gender diversity, the aetiology of gender identity has become a subject of increasing public interest. Prompted by the growing public debate, we review here the extant twin literature regarding the origins of gender diversity. Literature was reviewed systematically, searching Medline, Embase and PubMed databases. Studies were deemed eligible if they: (i) conducted a twin study, (ii) investigated gender identity or gendered behaviour, and (iii) provided an estimate of the magnitude of genetic or environmental contribution. After screening 290 non-duplicate titles and abstracts, 16 articles were included in the final review. Most eligible studies provided evidence of both genetic and environmental contributions to gender identity and gendered behaviour. For gendered behaviour, genetic contributions ranged from 0.10 to 0.77, non-shared environmental contributions ranged from 0.15 to 0.75, and shared environmental contributions ranged from 0.00 to 0.49. For gender identity, genetic contributions ranged from 0.00 to 0.84, non-shared environmental contributions ranged from 0.15 to 0.96 and shared environmental contributions ranged from 0.00 to 0.70. Given the variability in results and methodology between studies, the true magnitude of these contributions remains unclear. No consistent differences in contributions were identified between assigned males and assigned females. While there is also recent evidence that prenatal hormone exposure may contribute to gender identity, the overall evidence from the literature is inconsistent. Twin studies indicate both genetic and environmental contributions to gender diversity. These results are important to inform ongoing public debate in this area and highlight the complex interplay of both genetics and environment.

**Supplementary Information:**

The online version contains supplementary material available at 10.1007/s10519-025-10231-3.

## Introduction

The aetiological basis for gender diversity remains unknown. However, similar to other complex human traits (Polderman et al. 2015), the prevailing theory is that a complex interaction between genes and the environment collectively contribute to the formation of gender identity (Coleman et al. 2022). Studies using a classic twin study design have assessed the validity of this hypothesis by comparing rates of concordance for a transgender and other gender diverse (hereafter, trans) identity in monozygotic (MZ, identical) and dizygotic (DZ, non-identical) twins (Polderman et al. 2018) (see Table 1 for glossary of relevant terms). Since MZ twins share 100% of their DNA, compared to 50% on average in DZ twins, twin studies offer a powerful opportunity to examine questions about the heritable contribution to gender identity. In addition, using univariate models, twin data can also facilitate assessment of the relative contributions of environmental factors shared between each twin pair (e.g. intrauterine and family environment) and non-shared environmental factors (e.g. unique life experiences) (Neale and Cardon 1992).

**Table 1:** Relevant terms and definitions. (adapted from theStandards of Care for the Health of Transgender and Gender Diverse People, Version 8; Coleman et al. 2022; and the Guidelines for Psychological Practice with Transgender and Gender Nonconforming people, American Psychological Association 2015)

Term	Definition
Gender Diverse	An inclusive term for all non-cisgender individuals, i.e. individuals whose gender identity does not align with their sex assigned at birth. For example, non-binary, transgender men, transgender women, gender non-conforming individuals, etc.
Gender Dysphoria	A diagnostic term from the DSM-5 that refers to the incongruence between sex assigned at birth and experienced gender, as well as gender-related distress or discomfort. Not all trans people experience gender dysphoria.
Gender identity	An individual’s intrinsic sense of their own gender (i.e. male, female, non-binary, other).
Gendered behaviour	The ways in which a person enacts or expresses their gender identity within the context of culture and society. This can include mannerisms, behaviours, and expressions that may align with (or challenge) traditional concepts of masculinity and femininity.
Non-binary	A gender identity that falls outside the traditional male and female binary. While some non-binary individuals identify as transgender, others do not.
Opposite sex twins	A pair of twins who were assigned the opposite sex at birth (e.g. one assigned male and one assigned female). This classification is commonly used in twin research to group pairs for genetic and environmental comparisons.
Same sex twins	A pair of twins who were assigned the same sex at birth (e.g. both assigned females, or both assigned males). This classification is commonly used in twin research to group pairs for genetic and environmental comparisons.
Sex (assigned at birth)	The classification of an individual as male, female, or intersex at birth, based on the appearance of the external genitalia. This classification does not necessarily reflect a person’s gender identity. In this manuscript, we refer to this construct using the terms “assigned male” and “assigned female”, in line with current international standards.
Transgender	An umbrella term including people whose gender differs from what is typically expected for their sex assigned at birth. This term is similar to gender diverse; however, some find “transgender” more restrictive than “gender diverse”. For example, some individuals who identify as non-binary do not identify as transgender.
Trans	A commonly used shorthand for transgender and gender diverse individuals.
Transsexual(ism)	A term historically used in clinical contexts, particularly in the International Classification of Diseases (ICD-9 and ICD-10) until 2022. While outdated and less commonly used today, some individuals self-identify with this term.

One environmental-based theory to have emerged from the recent twin literature relates to a possible aetiological role for sex hormone exposure *in utero*. This theory has its origins in previous studies of congenital adrenal hyperplasia, a rare condition that exposes the developing foetus to high levels of masculinising steroid hormones. Genetically female individuals (i.e. those carrying an XX karyotype) with congenital adrenal hyperplasia have been observed to have higher than expected rates of male gender identity (Meyer-Bahlburg et al. 2006; Pasterski et al. 2015; Ristori et al. 2020). In this way, prenatal brain exposure to different levels of masculinising hormone activity has been proposed to contribute to gender identity development (Hines 2011a, b; Ristori et al. 2020). More recently, twin studies have provided further evidence to support this theory by comparing the concordance of gender diversity in same- and opposite-sex twins (Karamanis et al. 2022), based on the hypothesis that opposite-sex twin pairs directly transfer sex hormones *in utero* and are thus exposed to different masculinising and feminising hormone profiles compared with those in same-sex twins (Ahrenfeldt et al. 2020; Miller 1994). However, it should be noted that, while there is some support for the notion of *in utero* twin sex hormone transfer in animals, the evidence in humans is conflicting (Ahrenfeldt et al. 2020).

Prompted by ongoing public debate about the origins of trans identities, the aim of this review is to evaluate the existing twin literature to explore evidence for genetic and environmental contributions to gender diversity, including possible sex hormone transfer. The conceptualisation of gender diversity has evolved substantially over recent decades, and previous literature has operationalised gender diversity in a variety of ways. To account for this and ensure a comprehensive review, we have examined a broad range of gender-related constructs, including both behavioural- and identity-based measures, as listed in Table 1.

## Methods

### Eligibility Criteria

The review was not registered with PROSPERO, as it was originally conceptualised as a narrative review (albeit with a systematic approach). Based on editorial advice, the review was subsequently adapted into a systematic review and followed relevant PRISMA guidelines (Page et al. 2021) unless otherwise stated. Inclusion criteria for the study comprised (i) a measurement of gender (i.e. gender identity, gender dysphoria, gender-related DSM diagnoses, masculinity and femininity, gender atypical or cross-gender behaviour); (ii) recruited twins or multiple birth offspring as participants or reviewed literature that did; (iii) reported information related to the size of genetic and/or environmental etiological contributions (or presented raw data enabling their calculation); and (iv) were written in English.

## Information Sources, Search Strategy and Selection Process

**Fig. 1:**
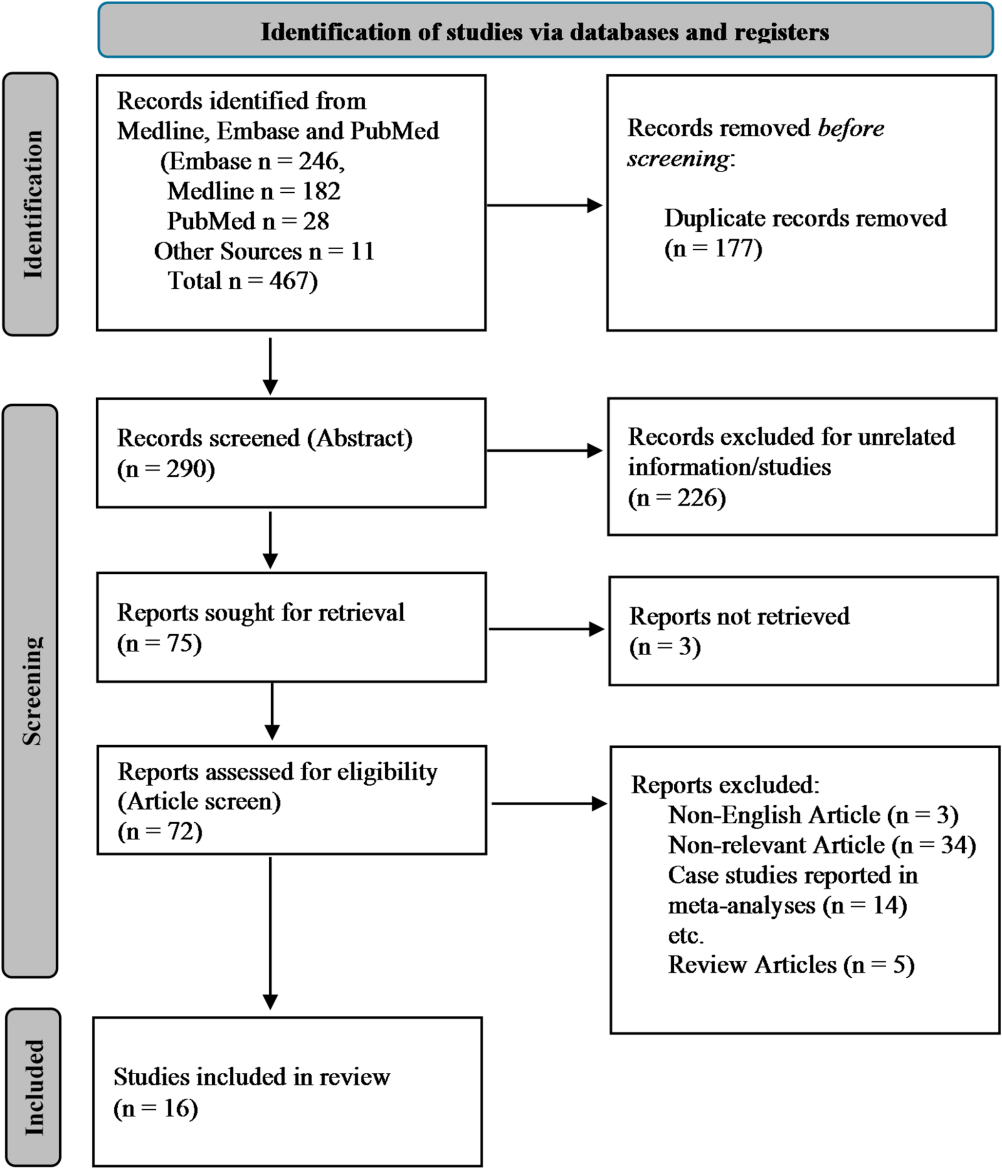
Flow chart of study selection

The search process was conducted by one reviewer (WC), performing searches of the Medline, Embase and PubMed databases. A flow diagram of the search process is presented in Fig. 1. Relevant Medical Subject Headings (MeSH) terms and keywords were used to search each database and included: (transgender/ or LGBT people/ or “transgender and gender nonbinary” or gender dysphoria/ or transsexualism/ or gender identity/), (etiology/ or genetics/ or phenotype/ or genome/ or environmental factor/), and (twins/ or triplets/ or multiple birth offspring/). A full description of the search strategy used for each database is provided in Supplementary Materials. Following removal of duplicates, 290 unique abstracts were initially screened against our study inclusion criteria, with 216 abstracts subsequently excluded based upon this screening. Of the remaining 74 abstracts, full text articles were able to be retrieved for 71. Of these, 15 articles met inclusion criteria. Snowballing from the 15 articles resulted in inclusion of one additional study, resulting in 16 articles in total for final review (Table 2).

## Data Collection Process and Study Category Determination

The data extraction process was conducted by two reviewers (WC and AR). Data extracted from the 16 articles consisted of the following seven variables: (i) participant birth assigned sex; (ii) participant age(s); (iii) relative contribution estimates from genetics, the shared environment between twins, and the non-shared environment between twins; (iv) concordance proportions between MZ and DZ twins and/or same sex (SS) and opposite sex (OS) twins; (v) the model used for study calculations; (vi) measures used to assess gendered behaviour and/or gender identity; and (vii) the recruitment method used in the study.

Articles were categorised into those whose research assessed gendered behaviour and/or gender identity, as determined by the measures used by each study. Studies were considered ‘gender behaviour (GB) research’ if their measure(s) assessed: (i) masculinity and femininity; and/or (ii) gender non-conformity, cross-gender behaviour or gender atypicality. Studies were considered ‘gender identity (GI) research’ if their measures assessed: (i) gender identity, or (ii) gender dysphoria or gender identity-related diagnoses. Several studies utilised multiple measures and were classified as both GB and GI research; for these studies, results are considered separately in the relevant sections below.

## Quality Assessment

The quality of each included study was assessed using a set of criteria adapted from Hayden et al. (2006) and Hayden et al. (2013), a process described by Ronald et al. (2021). The quality assessment scale evaluated four key domains: (i) study participation, (ii) outcome measurement, (iii) confounding, and (iv) statistical analysis and reporting. The two remaining criteria from the original scale (attrition and prognostic factor measurement) were excluded as they were not applicable to study design and/or topic of interest.

Study participation was separately evaluated across each of the following areas:

- There is adequate description of the key characteristics of the study population.
- The sampling frame and recruitment are described, including characteristics of the place of recruitment, or authors clearly reference where this information can be found.
- Inclusion and exclusion criteria are described, or authors clearly reference where this information can be found.

Outcome measurement was separately evaluated across each of the following areas:

- A clear definition of the outcome measure is provided.
- The outcome is operationalised in a way that adequately represents the outcome of interest.
- Indications are provided for the validity and reliability of the outcome measure, or a reference is provided.
- The method and setting of the outcome measurement is the same for all study participants.

Confounding was separately evaluated across each of the following areas:

- Potential confounders are accounted for in the analysis.
- Potential confounders are directly mentioned in-text as limitations.

Statistical analysis was separately evaluated across each of the following areas:

- There is sufficient presentation of the data to assess the adequacy of analytic strategy.
- The number of participants in the target sample supports sufficient statistical power.
- The selected statistical model is adequate for the design of the study.

Each study was evaluated independently by two reviewers (WC and AR), with conflicts resolved through discussion to reach consensus. Results are reported for each criterion individually for transparency.

## Synthesis Methods and Statistical Analyses

Point estimates of genetic, shared environmental and non-shared environmental contributions were typically provided within each paper. Where shared environmental and non-shared environmental contributions were not explicitly calculated by the original authors, these were left blank in our results. Where the genetic contribution was not explicitly stated by a paper, we were able to calculate it using the raw concordance data and the following equation (Falconer 1965), which provides an estimate of heritability based on the concordance proportion for MZ twins (R_MZ_) and DZ twins (R_DZ_):

H= 2×(R_MZ_−R_DZ_)

Confidence intervals (CIs) for point estimates of genetic contribution were provided by most papers. If not provided by the original authors, we calculated 95% confidence intervals from the raw concordance data using a z-test of two proportions where possible. All calculations were performed using STATA (version 16, StataCorp LLC). For studies lacking both reported CIs and raw data necessary for calculation, results were included in the Forest plots without corresponding CIs.

## Results

Eight twin studies assessed GB, five assessed GI, and three assessed both GB and GI. A summary of the characteristics of each study is presented in Table 2.

**Table 2:** Study characteristics – location, participant demographics, recruitment method and description of each study

Study	Study classification, location, and number of participants (*N*)	Assigned sex composition of twin pairs	Age (Range, Median or Mean)	Zygosity	Recruitment Method	Description of study	Measures used
Alanko et al. (2009)	GB Finland *N* = 3,593 individuals	Both assigned male and assigned female pairs.	Range: 33–43 years of age	• 91 assigned male MZ pairs • 247 assigned female MZ pairs • 110 assigned male DZ pairs • 270 assigned female DZ pairs • 203 opposite sex DZ pairs • 207 assigned male MZ individuals • 199 assigned female MZ individuals • 329 assigned male DZ individuals (from same-sex pairs) • 395 single assigned female DZ twins (from same-sex pairs) • 289 (95 assigned male and 194 assigned female) individuals from opposite-sex pairs.	Data from the Central Population Registry of Finland	Retrospective twin study on childhood gender atypical behaviour and sexual orientation. This study analysed the effects of genetics on each trait and the phenotypic association between the two variables.	Likert scale from 1–5 assessing gender role behaviours (*n* = 13) developed from the Recalled Childhood Gender Identity/Gender Role Questionnaire by Zucker et al. (2006).
Bailey et al. (2000)	GB and GI Australia *N* = 4,901 individuals	Both assigned male and assigned female pairs.	Median: 29 years	• 312 assigned male MZ • 182 assigned male DZ • 668 assigned female MZ • 376 assigned female DZ • 353 opposite-sex DZ pairs	Australian National Health and Medical Research Council Twin Register (ATR).	Cross-sectional and retrospective twin study on sexual orientation, childhood gender non-conformity and continuous gender identity. This study used uni- and multi-variate analyses to determine contributions from genetics, the shared environment (between twins) and the non-shared environment.	Gender non-conformity was a retrospective measure developed from several published scales. Gender identity was a cross-sectional measure based on 7 items taken from Finn (1987), with each question measured on a 7-point Likert scale.
Buhrich et al. (1991)	GB and GI Australia *N* = 158 pairs	Assigned male pairs only.	Median: 25 years	• 95 assigned male MZ pairs • 63 assigned male DZ pairs.	Australian NH & MRC Twin Registry	Cross-sectional and retrospective twin study on sexual orientation, sexual identity, and sex-dimorphic behaviours in assigned males. This study used uni- and multi-variate analyses to determine the genetic and environmental influences for each trait.	Sex-dimorphic behaviours and gender identity were measured using a questionnaire developed by McConaghy et al. (1979). Sex-dimorphic questions relayed to retrospective childhood sex-typed behaviours. Childhood gender identity was measured by whether the participant “ever wished to be of the opposite sex”. Adult gender identity was measured using four questions identity-related questions.
Burri et al. (2011)	GB and GI United Kingdom *N* = 4,426 individuals	Assigned female pairs only.	Mean = 53.36 years	• 906 assigned female MZ pairs • 806 assigned female DZ pairs • 642 assigned female individual twins	“Twins UK” Registry	Cross-sectional and retrospective twin study on female sexual orientation, childhood gender typicality and adult gender identity. This study used uni- and multi-variate analyses to determine the genetic and environmental influences for each of the three traits.	Gender typicality was measured using a scale of 4 items assessing retrospective childhood sex-typed behaviour. Adult gender identity was a cross-sectional measure based on 7 items similar to that used in Bailey et al. (2000) with each question measured on a 7-point Likert scale.
Coolidge et al. (2002)	GI Global (Internet) *N* = 314 pairs.	Both assigned male and assigned female pairs.	MZ Mean (SD): 9.4 (3.4) years DZ Mean (SD): 10.1 (3.6) years	• 44 assigned male MZ pairs • 52 assigned female MZ pairs • 20 assigned male DZ pairs • 20 assigned female DZ pairs • 21 O/S DZ pairs	Participants were the parents of twins. Recruited through internet and local newspaper advertisements.	Cross-sectional parent-reported twin study on Gender Identity Disorder (GID). The study used univariate modelling to calculate the heritability of GID.	GID was measured using a six-item scale based on the DSM-IV, created by the study researchers.
Diamond (2013)	GI Global N (Meta-analysis) = 43 pairs N = (Survey) = 69 pairs	Both assigned male and assigned female pairs.	Not stated, adult.	• 39 assigned male MZ pairs • 21 assigned male DZ pairs • 35 assigned female MZ pairs • 17 assigned female DZ pairs	Two recruitment methods: meta-analysis and survey. Sources obtained from published or otherwise available media (e.g., TV, Web, or film) in which “transsexual twins” are mentioned.	Literature search and a survey, combined to report the concordance rates for a transgender identity in MZ and DZ twins.	For participants of the survey, gender identity was measured using self-report.
Heylens et al. (2012)	GI Global *N* = 43 pairs	Both assigned male and assigned female pairs.	Not stated	• 8 assigned female MZ pairs • 5 assigned female DZ pairs • 15 assigned male MZ pairs • 15 assigned male DZ pairs	Meta-analysis of case study literature. Additional pairs were included from hospital data (Ghent University Hospital and Toronto Centre for Addiction and Mental Health).	Systematic literature review of the case study literature into transgender twins and additional case reports from hospital records. Calculated concordance rates for MZ and DZ twins from the published case studies.	Not defined.
Iervolino et al. (2005)	GB England and Wales *N* = 3,990 individuals	Both assigned male and assigned female pairs.	Range: 3–4 years old	• 906 assigned male MZ pairs • 1,022 assigned female MZ pairs • 963 assigned male DZ pairs • 903 assigned female DZ pairs • 90 assigned male sibling pairs • 106 assigned female sibling pairs	Data from the Twin Early Development Study (TEDS).	Cross-sectional parent-reported twin study on sex-typed behaviour in preschool children. The study used univariate modelling to calculate genetic, shared environmental, twin-specific environmental and non-shared environmental contributions to sex-typed behaviour. Twin-specific environment from comparisons between DZ twins and non-twin siblings.	Sex-typed behaviour was measured using the Preschool Activities Inventory (PSAI) from Golombrok & Rust, 1993). This scale comprises 24 items with a 5-point Likert scale.
Karamanis et al. (2022)	GI Sweden *N* = 67 pairs	Both assigned male and assigned female pairs.	Range: 11.2 to 64.2 years	Not measured, assigned sex: • 27 O/S pairs • 13 assigned male pairs • 27 assigned female pairs	Recruited participants over 10 years old through the National Patient Register of Sweden.	Investigated genetic and environmental effects on gender identity through a study of same-sex and opposite-sex twin pairs. Measured concordance rates between all twins with a GD diagnosis from the Swedish Register.	Individuals with 4 + diagnoses of Gender Dysphoria (or 1 + diagnosis and 1 + medical intervention for GD) were eligible.
Knafo et al. (2005)	GB United Kingdom *N* = 5112 pairs	Both assigned male and assigned female pairs.	Range: 3–4 years old	• 1,362 assigned male MZ pairs • 1,427 assigned male DZ pairs • 1,564 assigned female MZ pairs • 1,446 assigned female DZ pairs	Twins’ Early Development Study (TEDS) born in the UK.	Cross-sectional parent-reported twin study on sex-typed behaviour. This study determined atypicality through statistical cut-offs (e.g. top 5%, top 10% etc.). Twins where at least one was determined atypical were investigated for concordance with their twin.	Masculinity and femininity were measured using the Preschool Activities Inventory (PSAI) from Golombok & Rust, 1993). This scale comprises 24 items with a 5-point Likert scale.
Lippa and Hershberger (1999)	GB United States *N* = 839 pairs	Both assigned male and assigned female pairs.	Age referred to as “college age”	• 219 assigned male MZ pairs • 135 assigned male DZ pairs • 293 assigned female MZ pairs • 195 assigned female DZ pairs	Twins who completed the United States National Merit Test in 1962 (*n* = 1507 pairs).	Cross-sectional twin study on gendered traits. Masculine instrumentality, feminine expressiveness and gender diagnosticity (the likelihood of being male or female based on alignment with gendered traits) were compared between MZ and DZ twins. Intraclass correlations were calculated to calculate genetic and environmental contributions to these traits.	Measured using the Adjective Check List (ACL) and California Psychological Inventory (CPI).
Loehlin et al. (2005)	GB Swedish and Australian adults, U.S. elderly, and Australian adolescents. Swedish pairs (*N* = 362), US elderly (*N* = 3787), Australian adults (*N* = 5672), Australian adolescents (*N* = 4119)	Both assigned male and assigned female pairs.	Swedish adults: Mean = 43.1 years United States elderly: Mean = 66.7 Australian adults: Mean = 42.2 Australian adolescents: Range = 12–16 years	• Swedish zygosity data not reported. • US elderly (143 assigned male MZ, 592 assigned female MZ, 61 assigned male DZ, 277 assigned female DZ) • Australian adults (383 assigned male MZ, 863 assigned female MZ, 215 assigned male DZ, 497 assigned female DZ) • Australian teenage (369 assigned male MZ, 384 assigned female MZ, 332 assigned male DZ, 310 assigned female DZ).	Samples obtained from prior studies, Jonsson et al. (2001), Loehlin et al. (1999), Stallings et al. (1999) and Wright & Martin (2004).	Cross-sectional data from questionnaires taken in prior studies that have included questions on gendered behaviour. Investigated gender diagnosticity (likelihood of being male or female based on a gendered trait) in four different samples. Calculated effects of genetics, shared environment and non-shared environment.	Swedish participants completed the Karolinska Scales of Personality questionnaire (KSP) from Schalling et al. (1987). The US Elderly participants completed an English version of the KSP. Australian adults completed the Eysenck Personality Questionnaire (EPQ) from Eysenck et al. (1985) and Cloningers Tridimentional Personality Questionnaire (TPQ) from Cloninger et al. (1991). Australian adolescents completed the Junior Eysenck Personality Questionnaire (JEPQ) from Eysenck et al. (1975).
Mitchell et al. (1989)	GB United States *N* = 140 pairs	Both assigned male and assigned female pairs.	Mean age = 11.1 years (SD 1.7 years).	• 13 assigned male MZ pairs • 25 assigned female MZ pairs • 9 assigned male DZ pairs • 14 assigned female DZ pairs • 9 O/S DZ pairs	Twin register at the University of Southern California and through local radio and newspaper advertisements.	Cross-sectional twin study with self-reported masculinity and femininity measures. The study calculated the genetic and environmental factors contributing to these traits.	Masculinity and femininity were measured with the Children’s Personality Attributes Questionnaire (CPAQ) by Hall and Halberstadt (1980) and the Adolescent Self-Perception Inventory (ASPI) by Thomas (1983).
Sasaki et al. (2016)	GI Japan *N* = 3332 pairs (*n* = 336 included in final analyses)	Both assigned male and assigned female pairs.	Range: 3–26 years	Total(s): • 1265 assigned female MZ pairs • 937 assigned male MZ pairs • 617 assigned female DZ pairs • 513 assigned male DZ pairs Concordant and Discordant pairs: • 4 assigned male children MZ • 4 assigned male children DZ • 15 assigned male adolescents MZ • 3 assigned male adolescents DZ • 8 assigned male adults MZ • 5 assigned male adults DZ • 13 assigned female children MZ • 12 assigned female children DZ • 76 assigned female adolescents MZ • 34 assigned female adolescents DZ • 46 assigned female adults MZ • 25 assigned female adults DZ • 12 O/S child pairs • 67 O/S adolescent pairs • 12 O/S adult pairs	Identified from the residential registry of local governments as pairs of individuals with the same date of birth and home address. Approximately 21,600 pairs were selected at random and were mailed a questionnaire.	Cross-sectional parent-reported twin study measuring traits of Gender Identity Disorder (from the DSM-IV). The study calculated genetic and environmental contributions to the traits, as well as directly addressing the Prenatal Hormone aetiological theory. This study also provided estimates of genetic and environmental stratified by (1) sex assigned at birth; and (2) age group.	The measure used was created for the present study based on the DSM-IV criteria for Gender Identity Disorder, with each question (*n* = 4) having a 6-point Likert scale.
van Beijsterveldt et al. (2006)	GB Netherlands Age 7 *N* = 7,526 pairs Age 10 *N* = 4,538 pairs	Both assigned male and assigned female pairs.	Range: 7–10 years old.	• 2430 assigned male MZ pairs • 2790 assigned female MZ pairs • 2477 assigned male DZ pairs • 2303 assigned female DZ pairs • O/S DZ pairs: 2295 (assigned male); 2303 (assigned female)	Netherland Twin Register.	Parent-reported twin study on cross-gender behaviour across two time points (age 7 and 10). The study operationalised cross-gender as either (1) behaving like; or, (2) wishing to be the opposite sex. The study calculated the magnitude of contribution to the trait from additive genetic factors, non-additive genetic factors and unique environmental factors.	Cross-gender behaviour was measured using two questions from the CBCL (Dutch Version) from Achenbach (1991) on a 3-point Likert scale.
Verweij et al. (2016)	GB Sweden *N* = 9520 participants	Both assigned male and assigned female pairs.	Range: 27–54 years Mean = 40.7 years	• 695 assigned female MZ pairs • 374 assigned male MZ pairs • 392 assigned female DZ pairs • 248 assigned male DZ pairs • 536 O/S DZ pairs • 5030 individuals from twin pairs	STAGE Cohort (32,000 Swedish twins born between 1959 and 1985).	Cross-sectional twin study on masculinity and femininity. This study investigated the role that genetics, environment and prenatal hormone exposure may play in these two traits.	Masculinity and femininity were measured using the Big Five Inventory (John et al. 1991). This comprised a 5-point Likert scale in all 44 items.

## Gendered Behaviour

**Fig. 2:**
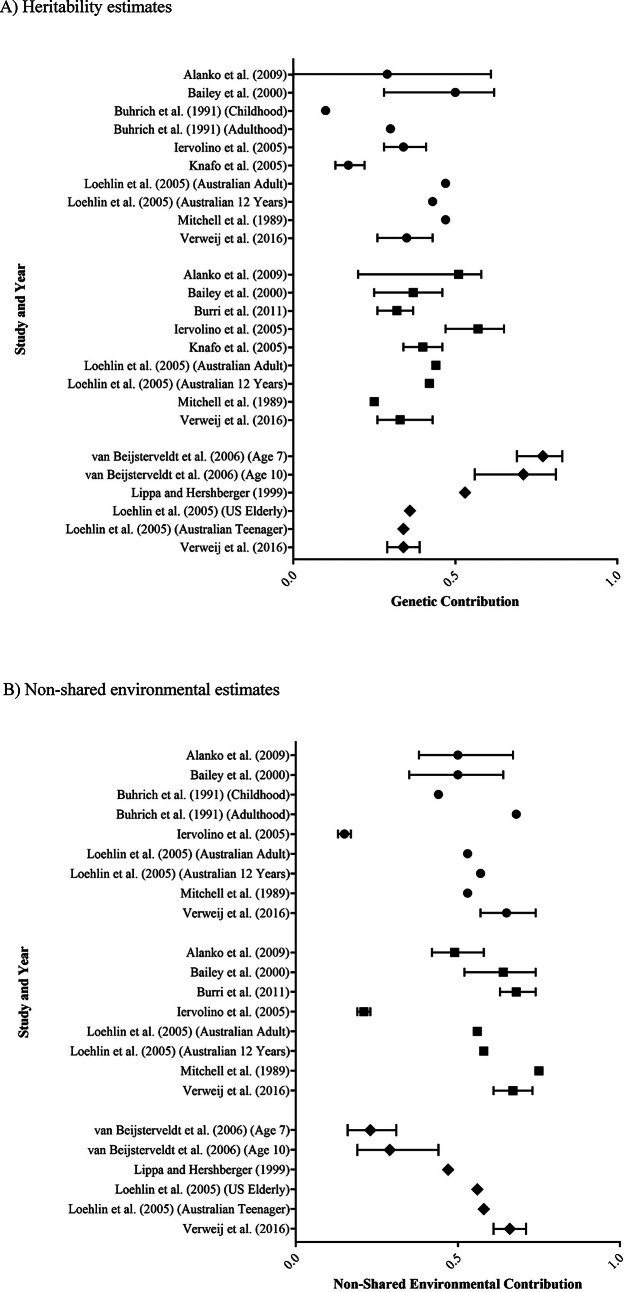

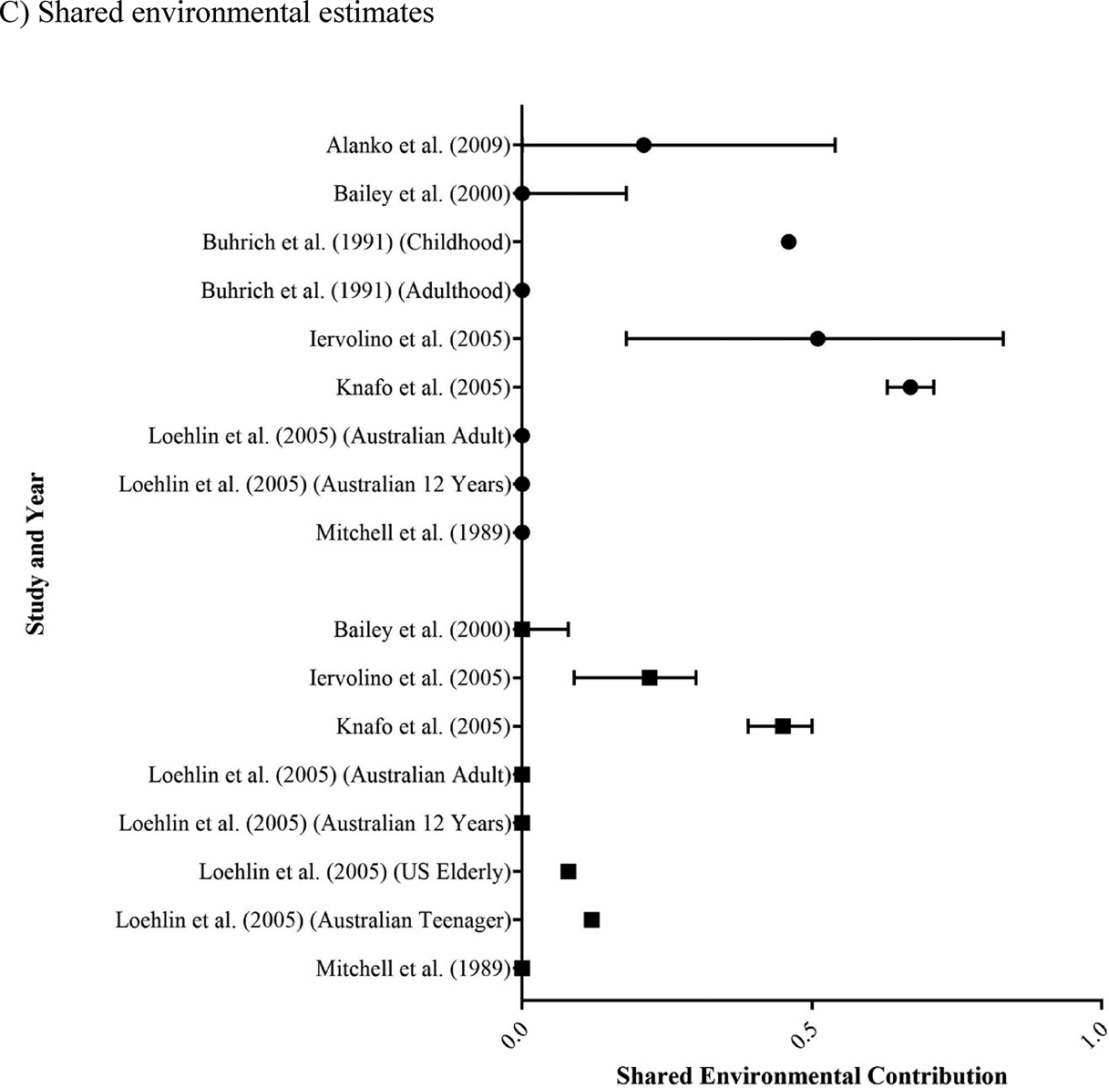
Forest plots of **A** heritability, **B** non-shared environment, and **C** shared environment estimates for gendered behaviour. Study results are grouped into those with assigned male participants only (● symbols), assigned female participants only (■ symbols) or both (◆ symbols). Estimates are a proportion between 0 and 1, with 95% confidence interval bars shown. Where studies have separated estimates into sub-groups, each is represented separately.

The 11 studies assessing GB focused on individual adherence to stereotypical male and female behaviours, attitudes and traits, and included the following studies: Alanko et al. (2009), Bailey et al. (2000), Buhrich et al. (1991), Burri et al. (2011), Iervolino et al. (2005), Knafo et al. (2005), van Beijsterveldt et al. (2006), Lippa and Hershberger (1999), Loehlin et al. (2005), Mitchell et al. (1989) and Verweij et al. (2016). A summary of the findings from these studies is presented in Table 3; Fig. 2.

Genetic contribution estimates for GB ranged from 0.10 to 0.77 (Fig. 2a). Notably, all 11 studies had heritability point estimates > 0, consistent with there being a heritable component to gendered behaviour. Several studies examined assigned male and assigned female pairs separately and/or together (across the 11 studies, this gave rise to results for 25 different sub-groups). Estimates showed a similar distribution regardless of assigned sex (assigned male: 0.10 to 0.50; assigned female: 0.25 to 0.57). Overall, of the 15 sub-groups for which 95% confidence intervals were available, 14 of these intervals did not include zero, indicating a significant genetic influence on GB. However, there were also 11 additional sub-groups for which 95% confidence intervals were not available.

Non-shared environmental contribution estimates for GB ranged from 0.15 to 0.75 (Fig. 2b). These findings are consistent with there being a non-shared environmental component to gendered behaviour. There were also no major differences in the non-shared environmental contributions observed across assigned male (0.15 to 0.68) and assigned female (0.21 to 0.75) estimates.

Four studies did not include the shared environment in their model of best fit or did not report a shared environmental value (van Beijsterveldt et al. 2006; Burri et al. (2011; Lippa and Hershberger (1999; Verweij et al. (2016). Of the remaining studies, shared environmental contributions ranged from 0.00 to 0.67 (Fig. 2c), with no differences identified between assigned male (0.00 to 0.67) and assigned female (0.00 to 0.45) estimates. Two studies identified shared environmental contributions across all sub-groups (Knafo (2005) and Iervolino et al. (2005) and three reported a role of the shared environment in some sub-groups but not others (Alanko et al. 2009; Buhrich et al. (1991; Loehlin et al. (2005). The remaining two studies reported shared environmental contributions of 0 (Bailey et al. 2000; Mitchell et al. (1989).

The prenatal hormone transfer theory was specifically investigated in two studies (van Beijsterveldt et al. 2006; Verweij et al. (2016) by comparing SS and OS pairs. Verweij et al. (2016) reported some evidence for the theory, identifying higher masculinity scores in assigned females in OS pairs than those in SS pairs. However, van Beijsterveldt et al. (2006) did not report any difference in masculinity scores in assigned females across their OS and SS pair samples. Similarly, neither study identified a difference in cross-gender behaviour in assigned males when comparing SS and OS pairs.

**Table 3:** Summary of twin studies assessing gendered behaviour

Study	Assigned sex composition of twin pairs	Age sub-groups	Genetics (95% CI)	Shared Environment (95% CI)	Non-Shared Environment (95% CI)
Alanko et al. (2009)	Assigned Male	-	0.29 (0.00,0.61)	0.21 (0.00,0.54)	0.50 (0.38,0.67)
	Assigned Female	-	0.51 (0.20,0.58)	-	0.49 (0.42,0.58)
Bailey et al. (2000)	Assigned Male	-	0.50 (0.28,0.62)	0.00 (0.00,0.18)	0.50 (0.35,0.64)
	Assigned Female	-	0.37 (0.25,0.46)	0.00 (0.00,0.08)	0.64 (0.52,0.74)
Buhrich et al. (1991)	Assigned Male	Children	0.10	0.46	0.44
	Assigned Male	Adults	0.30	0.00	0.68
Burri et al. (2011)	Assigned Female	-	0.32 (0.26,0.37)	–	0.68 (0.63,0.74)
Iolino et al. (2005)	Assigned Male	-	0.34 (0.28,0.41)	0.51 (0.18,0.83)	0.15 (0.13,0.17)
	Assigned Female	-	0.57 (0.47,0.65)	0.22 (0.09,0.30)	0.21 (0.19,0.23)
Knafo et al. (2005)	Assigned Male	-	0.17 (0.13,0.22)	0.67 (0.63,0.71)	-
	Assigned Female	-	0.40 (0.34,0.46)	0.45 (0.39,0.50)	-
Lippa and Hershberger (1999)	Both	-	0.53	–	0.47
Loehlin et al. (2005)	Assigned Male	Children	0.43	0.00	0.57
	Assigned Female	Children	0.42	0.00	0.58
	Both	Adolescents	0.30	0.12	0.58
	Assigned Male	Adults	0.47	0.00	0.53
	Assigned Female	Adults	0.44	0.00	0.56
	Both	Elderly	0.36	0.08	0.56
Mitchell et al. (1989)	Assigned Male	-	0.47	0.00	0.53
	Assigned Female	-	0.25	0.00	0.75
van Beijsterveldt et al. (2006)	Both	Children (7 years)	0.77 (0.69,0.83)	-	0.23 (0.16,0.31)
	Both	Children (10 years)	0.71 (0.56,0.81)	-	0.29 (0.19,0.44)
Verweij et al. (2016)	Assigned Male	-	0.35 (0.26,0.43)	-	0.65 (0.57,0.74)
	Assigned Female	-	0.33 (0.26,0.39)	-	0.67 (0.61,0.73)
	Both	-	0.34 (0.29,0.39)	-	0.66 (0.61,0.71)

Contribution estimates are shown and represent a proportion between 0 and 1, where 0 indicates no contribution and 1 indicates complete contribution. The three potential contributions – genetics, shared environmental factors between twins, and non-shared environmental factors unique to each twin – are shown separately. Where all three contributing factors were investigated, their sum equals 1. Some studies only investigated one or two of these factors. For some studies, results were reported based on separate sub-groups and these are indicated where relevant.

## Gender Identity

**Fig. 3:**
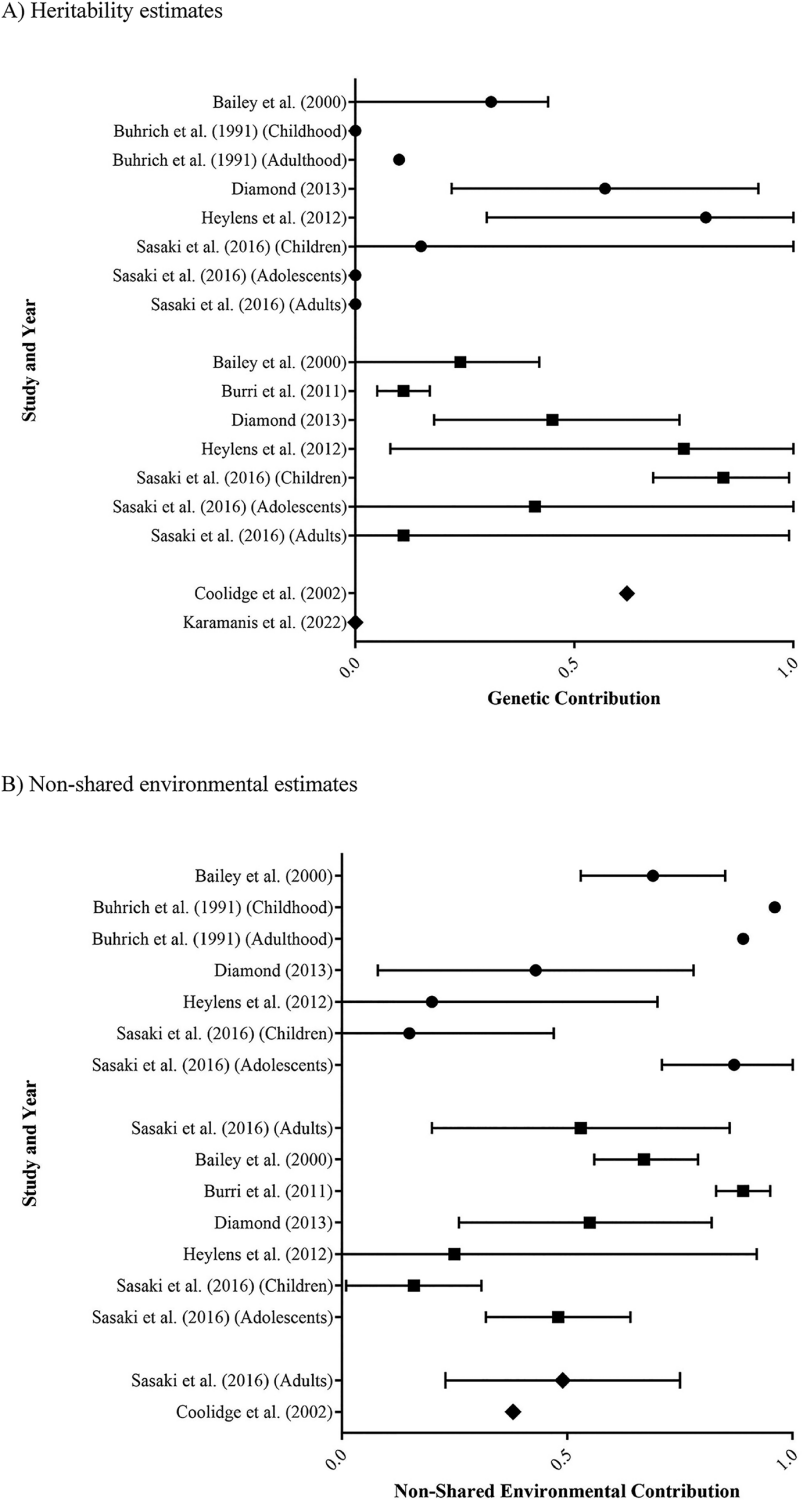

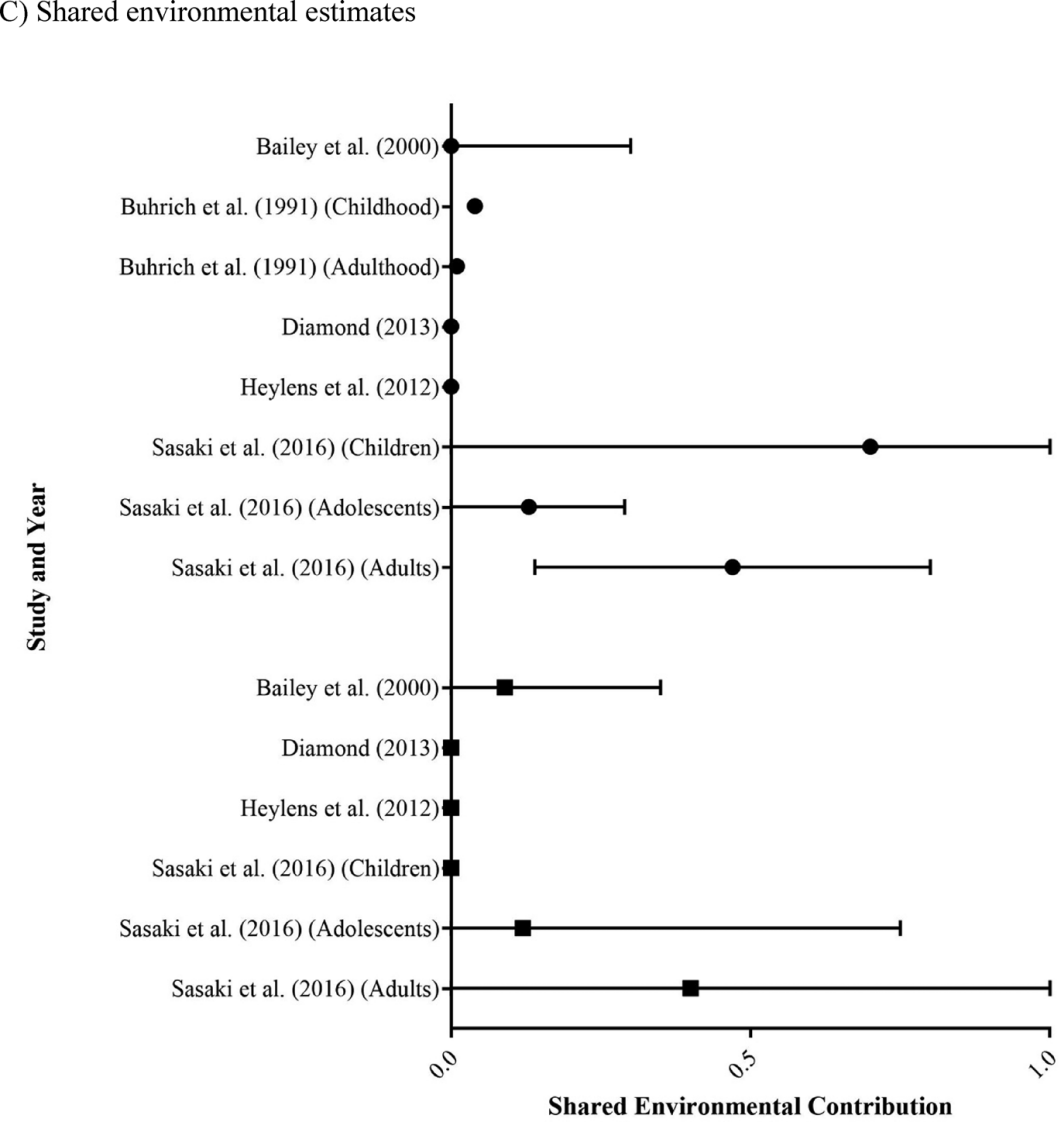
Forest plots of **A** heritability, **B** non-shared environment, and **C** shared environment estimates for gender identity. Study results are grouped into those with assigned male participants only (● symbols), assigned female participants only (■ symbols) or both (◆ symbols). Estimates are a proportion between 0 and 1, with 95% confidence interval bars shown. Where studies have separated estimates into sub-groups, each is represented separately.

Eight studies assessed the genetic and/or environmental influences on gender identity, included Bailey et al. (2000), Buhrich et al. (1991), Burri et al. (2011), Coolidge et al. (2002), Diamond (2013), Heylens et al. (2012), Karamanis et al. (2022) and Sasaki et al. (2016). A summary of findings from these studies is presented in Table 4; Fig. 3.

From the eight studies, heritability estimates for GI ranged from 0.00 to 0.84 (Fig. 3a). Notably, only one study reported an overall genetic contribution of 0 (Karamanis et al. 2022), while two additional studies reported heritability estimates of 0 in some sub-groups but not in others (Buhrich et al. 1991; Sasaki et al. (2016). No discernible difference was identified between assigned male (0.00–0.80) and assigned female (0.11–0.84) estimates. Overall, these results are consistent with there likely being a heritable component contributing to gender identity.

Non-shared environmental contribution estimates ranged between 0.15 and 0.96 (Fig. 3b). These findings are consistent with there being a non-shared environmental component to gender identity. No difference was observed between studies reporting separate assigned male (0.15–0.96) and assigned female (0.16–0.89) estimates, with both groups exhibiting a wide range of variability.

While estimates of shared environmental contributions ranged from 0.00 to 0.70 (Fig. 3c), four of the five studies to look at this were within a much narrower range of 0.00 to 0.09 (Bailey et al. 2000; Buhrich et al. (1991; Diamond (2013; Heylens et al. (2012). The remaining study, Sasaki et al. (2016) reported a broad spread of shared environmental contributions across the six sub-groups (stratified by age group and assigned sex), with upper limits of 0.70 for assigned males and 0.40 for assigned females (although both of these estimates had an associated 95% confidence interval of 0.00–1.00).

Two studies on gender identity directly addressed the prenatal hormone transfer theory (Karamanis et al. 2022; Sasaki et al. (2016); however, their evidence was conflicting. The former reported a higher concordance in OS pairs, with no concordant pairs in the SS group. In contrast, the latter observed no differences between the OS and SS pairs for both assigned males and assigned females.

**Table 4:** Summary of twin studies assessing gender identity

Study	Assigned sex composition of twin pairs	Age sub-groups	Number of pairs analysed	Genetics (95% CI)	Shared Environment (95% CI)	Non-Shared Environment (95% CI)
Bailey et al. (2000)	Assigned Male	*-*	Not specified	0.31 (0.00,0.44)	0.00 (0.00,0.30)	0.69 (0.53,0.85)
	Assigned Female	-	Not specified	0.24 (0.00,0.42)	0.09 (0.00,0.35)	0.67 (0.56,0.79)
Buhrich et al. (1991)	Assigned Male	Children	*n* = 158	0.00	0.04	0.96
	Assigned Male	Adults		0.10	0.01	0.89
Burri et al. (2011)	Assigned Female	-	Not specified	0.11 (0.05,0.17)	-	0.89 (0.83,0.95)
Coolidge et al. (2002)	Assigned Both	-	*n* = 157	0.62	-	0.38
Diamond (2013**)**	Assigned Male	-	*n* = 33	0.57 (0.22,0.92)	0.00	0.43 (0.08,0.78)
	Assigned Female	-	*n* = 36	0.45 (0.18,0.74)	0.00	0.55 (0.26,0.82)
Heylens et al. (2012)	Assigned Male	-	*n* = 31	0.80 (0.30,1.00)	0.00	0.20 (0.00,0.70)
	Assigned Female	-	*n* = 13	0.75 (0.08,1.00)	0.00	0.25 (0.00,0.92)
Karamanis et al. (2022)	Both	-	*n* = 67	0.00*	-	-
Sasaki et al. (2016)	Assigned Male	Children	*n* = 8	0.15 (0.00, 1.00)	0.70 (0.00, 1.00)	0.15 (0.00, 0.47)
	Assigned Male	Adolescents	*n* = 18	0.00 (0.00,0.00)	0.13 (0.00, 0.29)	0.87 (0.71, 1.00)
	Assigned Male	Adults	*n* = 13	0.00 (0.00,0.00)	0.47 (0.14, 0.80)	0.53 (0.20, 0.86)
	Assigned Female	Children	*n* = 25	0.84 (0.68, 0.99)	0.00 (0.00, 0.00)	0.16 (0.01, 0.31)
	Assigned Female	Adolescents	*n* = 110	0.41 (0.00,1.00)	0.12 (0.00, 0.75)	0.48 (0.32, 0.64)
	Assigned Female	Adults	*n* = 71	0.11 (0.00,0.99)	0.40 (0.00, 1.00)	0.49 (0.23, 0.75)

* Study did not actually measure zygosity; therefore, estimate is inferred from results. Contribution estimates are shown and represent a proportion between 0 and 1, where 0 indicates no contribution and 1 indicates complete contribution. The three potential contributions – genetics, shared environmental factors between twins, and non-shared environmental factors unique to each twin – are shown separately. Where all three contributing factors were investigated, their sum equals 1. Some studies only investigated one or two of these factors. For some studies, results were reported based on separate sub-groups and these are indicated where relevant. The number of pairs analysed represents the number of pairs where at least one co-twin was classified as trans in the study, when not stated in the paper, these have been reported as “not specified”

### Quality Assessment

Most studies performed well in the quality assessment (Table 5), with the majority fulfilling > 50% of the criteria. Key strengths across the studies included detailed descriptions of study populations and recruitment processes, as well as acknowledgement of potential confounders. Notably, studies examining gendered behaviour (or both concepts) tended to perform better in the quality assessment compared to those investigating gender identity specifically. While nearly all studies included criteria that could not be definitively classified as a strict “yes” or “no”, only four studies failed to meet two or more criteria. These findings reflect an overall high quality across the included studies.

**Table 5:**
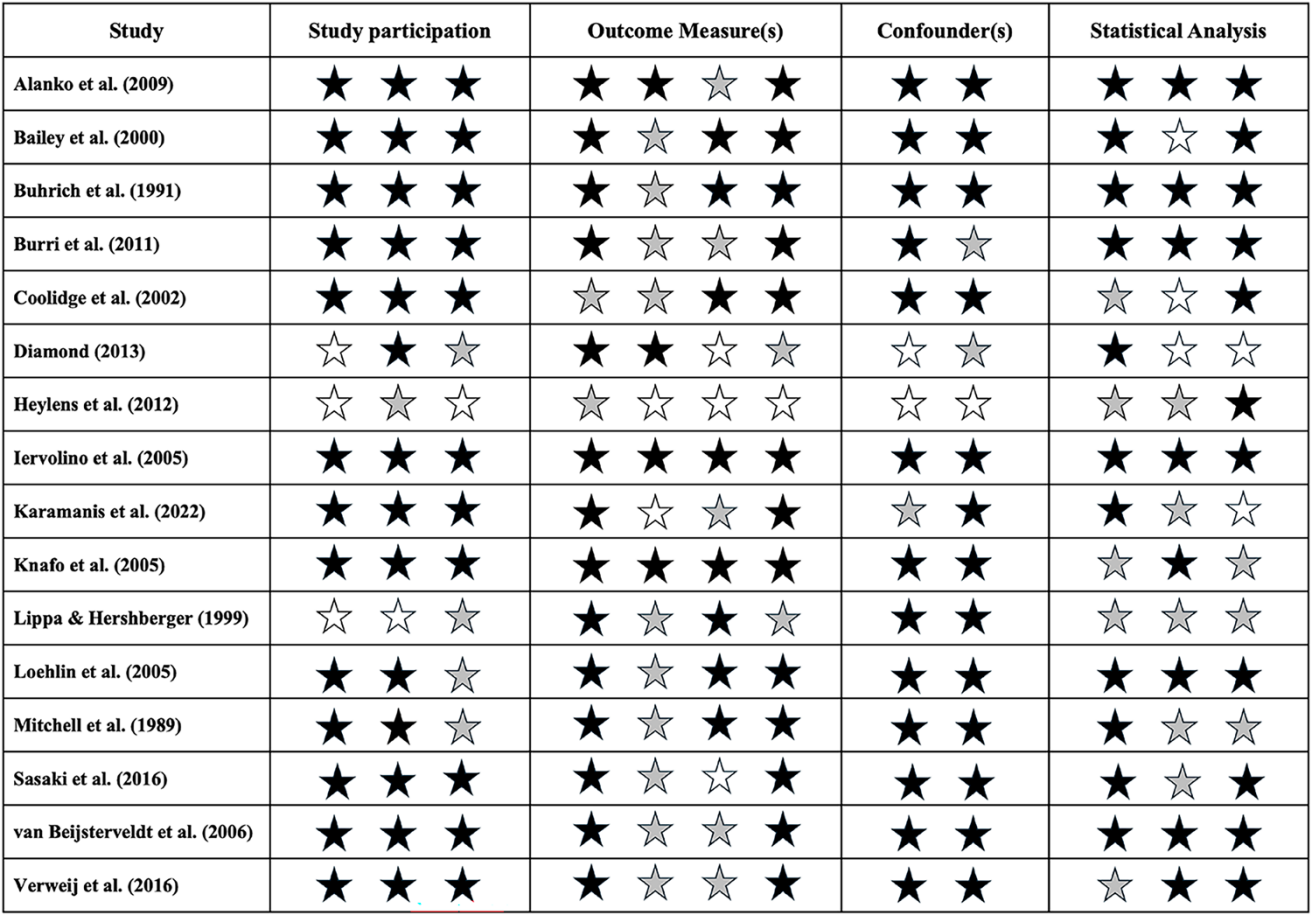
Quality assessment of included twin studies

The quality assessment results are presented for each criterion, grouped into the four broader categories of study participation, outcome measures, confounders, and statistical analysis. The order of stars corresponds to the sequence of criteria outlined in the Methods (Quality Assessment subsection), with each criterion represented by a star. A black star indicates the criterion was fulfilled, a white star indicates the criterion was not fulfilled, and a grey star denotes insufficient information or situations where the criterion could not be strictly answered as “fulfilled” or “not fulfilled”. 

## Discussion

Taken together, our review of the twin literature suggests the existence of heritable and environmental contributions to both gendered behaviour and gender identity, but data pertaining to a possible role for prenatal hormone transfer were scarce and conflicting.

All reviewed studies provided evidence supporting a genetic contribution to GB. Heritability estimates for most complex human behaviours typically fall between 0.30 and 0.60 (Polderman et al. 2015), and most point estimates for GB similarly clustered within this range (Fig. 2a). Despite evidence supporting a heritable influence on GB, the exact magnitude of the contribution remains ill-defined, given the wide distribution of estimates and associated confidence limits (Fig. 2a). This variability is likely to be at least partially explained by heterogeneity in study design (retrospective or cross-sectional), type of reporting (parent- or self-report), and, importantly, the measures chosen to operationalise gendered behaviour, as almost every study employed a different measure (Table 1).

There was also evidence of a genetic contribution to GI, which is consistent with recent molecular genetic studies that identified variants in sex hormone signalling genes which may predispose individuals to a trans identity (Foreman et al. 2019; Theisen et al. 2019). However, compared to GB, there were fewer studies assessing GI and heritability estimates were more varied. Previous reports based at least in part on the case study literature tended to yield large heritability estimates (Heylens et al. 2012; Diamond 2013). However, some have argued that these estimates are likely to be skewed given the potential bias of the case study literature to identify concordant pairs (Karamanis et al. 2022). To minimise the risk of ascertainment bias, a register-based approach was utilised by Karamanis et al. (2022) and interestingly their study was the only one to find no evidence of genetic involvement. However, there are two important caveats to note when interpreting these authors’ results. Firstly, although the authors did not identify any concordant pairs within their sample of 40 same-sex twins, a key limitation of the study was its failure to measure zygosity. As a result, the study could not distinguish between MZ and DZ same-sex twins. Their heritability estimate of 0 is therefore based on the assumption that at least some of their pairs were MZ and, while this is a reasonable assumption, it is not possible to assess whether the underlying number of MZ pairs provided sufficient statistical power to properly investigate heritability. In other words, their heritability estimate of 0 could be due to a type 2 statistical error arising from having too small a MZ sample (i.e. false negative result). Secondly, the methods by which these authors operationalised gender diversity are likely to have excluded many trans individuals from consideration. Specifically, the study used a Swedish national registry to identify individuals over the age of 10 years who had received at least four diagnoses of gender dysphoria or at least one diagnosis followed by gender-affirming treatment. In doing so, the authors identified 2592 trans individuals altogether. However, based upon a previously published incidence rate of around 1% for trans individuals within the Swedish population (Åhs et al. 2018), one would expect a sample size of 92,500 transgender individuals based on census data (Statistics Sweden 2024). This suggests that the methods used by Karamanis et al. likely led to the exclusion of the majority of trans individuals and was thus also subject to bias.

All reviewed studies also provided evidence for non-shared/unique environmental contributions to both GB and GI (Figs. 2b and 3b). These results are consistent with most complex human behaviours, which have both genetic and unique environmental contributions (Polderman et al. 2015). However, from the study designs used, it is impossible to determine whether these unique environmental factors are non-shared prenatal exposures, non-shared postnatal factors, or a combination. Additionally, there is also great heterogeneity in the effect sizes reported, likely due to both measurement and random error, making it difficult to determine an accurate estimate for the contribution from unique environmental experiences. Similar to the genetic results, this is likely the consequence of having small sample sizes for many reported estimates, resulting in large confidence intervals, particularly in the GI estimates (Fig. 3b). In considering our results, it is important to note that these findings do not provide support for or against any specific non-shared environmental influences. After all, the twin studies that we reviewed were not designed to interrogate the role of specific environmental factors. Thus, our data cannot comment on the validity of the various theories, such as social contagion (Diaz & Bailey 2023; Littman 2018), that have been proposed in this realm in recent years.

In contrast to the findings identified in the genetic and non-shared environmental results, shared environmental influences were often found to have no aetiological role. Across the GB studies, there were only two studies that consistently reported an overall shared environmental contribution, Iervolino et al. (2005) and Knafo et al. (2005). It is important to note that these two studies had overlapping samples, with both using the Twin Early Development Study (TEDS) dataset, comprising parental responses about their 3- and 4-year-old children. Interestingly, Iervolino et al. (2005) points out that sibling influences (which would comprise part of the shared environment) may be amplified in twins at this young age, due to parent reports of mostly group play and shared toys/interests. This underscores the importance of differentiating the GB literature from the GI literature, especially given early life gender nonconformity does not necessarily predict a transgender identity later in life (Steensma et al. 2013).

Only two studies, Sasaki et al. (2016) and Karamanis et al. (2022), reported substantial evidence of contributions from the shared environment on GI. However, for Sasaki et al. (2016), 95% confidence intervals for all but one of their non-zero sub-group estimates included 0, thus providing minimal evidence of a shared environmental contribution. Consistent with this, these authors concluded that their findings did not provide support for the prenatal hormone transfer theory. In contrast, Karamanis et al. (2022) suggested that the shared prenatal environment was a significant contributor to gender identity through their comparison of SS twins, OS twins and non-twin siblings. Bearing in mind that OS twin pairs and non-twin siblings are conceptually the same from an inheritance viewpoint (with an average of 50% shared DNA), it was notable that the concordance rate among OS twin pairs (37%) in their study was much greater than among non-twin siblings (0.16%). However, if prenatal hormone transfer was aetiologically important, we likely would have identified larger contributions from the shared environment across all other studies (Fig. 3c), but this was not the case. In this way, twin studies overall do not support a role for prenatal hormone transfer in the development of gender diversity.

Taken together, the results of this systematic review have important implications for the current public discourse regarding the aetiology of gender diversity. Notably, evidence regarding the genetic contribution towards gender diversity has been increasingly ignored in public discussion. Instead, theories regarding environmental causes have been promoted, often with little to no evidentiary basis. This approach is best exemplified in the Cass Review, a highly influential 2024 report on paediatric gender identity services operated by the National Health Service in England (Cass 2024). In this report, results of past twin studies that suggested a genetic contribution to gender diversity were largely discounted, and instead emphasis was placed on the findings of Karamanis et al. (2022), which as noted above were an outlier and had key methodological limitations. At the same time, Cass proposed other theories related to various environmental exposures, including social media and pornography, whose links to gender diversity lack any empirical support. Looking ahead, it is critical that discussions regarding the aetiology of gender diversity take into account the findings of our systematic review which indicate that the majority of twin studies in this area were of high quality and consistently indicate that a genetic contribution to gender diversity exists.

This review has identified multiple limitations and knowledge gaps within the existing twin literature, and which together provide a roadmap for future studies in this area. First, existing estimates of heritability are based on heterogenous methods of assessing GB and GI. These major differences hinder the possibility of meta-analysis and thus prevent calculation of heritability estimates with any confidence in this review. Second, future studies should rely on data provided directly by participants about their own gender identity, preferably as quantitative estimates that take account of non-binary categories (Twist and de Graaf 2019), which would increase the accuracy of studies assessing the relative contributions of heritable and environmental influences shaping the formation of GI. Third, researchers should ensure systematic recruitment in future research, ensuring representative samples and meaningful estimates. Fourth, the social and cultural context of study participants is likely to influence their expression of gender diversity. For example, stigma and/or lack of acceptance regarding gender diversity is likely to lead to suppression of transgender identities and, in doing so, lead to higher estimates of environmental contributions. We were unable to determine this context for the individual studies included in our review, but given that the studies were undertaken across a broad range of different countries and time periods, it seems highly probable that the contexts varied considerably, and might explain some of variability in the reported estimates for genetic and environmental contributions. Future studies should therefore bear this in mind. Finally, twins often share an extremely close relationship (Tancredy and Fraley 2006), and it would be interesting to explore how one twin coming out as trans may influence the twin dynamic. Future qualitative interviews could be used to provide insights into this phenomenon of being both a twin and trans.

While this review was able to identify valuable insights into the origins of GB and GI, it is also subject to several limitations. First, we would have liked to have performed a meta-analysis, but the heterogeneity of study designs, measurements, and sample characteristics (Table 1) made this impractical. Second, while considering both GB and GI was valuable, it is important to acknowledge that these are distinct constructs with potentially separate aetiological contributions. Further, some studies included in this review examined broad concepts of gender that made it challenging to discern whether they were measuring behaviour, identity, or both. Related to this, recognising that GB in childhood does not necessarily predict GI later in life is crucial for accurate interpretation of the findings. Third, some of the featured studies included assessment of sexual attraction and/or orientation (Alanko et al. 2009; Bailey et al. 2000; Buhrich et al. 1991; Burri et al. 2011), but these results were not summarised here, since we felt that sexual attraction and/or orientation are separate constructs and thus beyond the scope of this review. Fourth, an individual’s genotype can influence their environmental exposures, giving rise to gene–environment correlations (rGE) (Plomin et al. 1977). In the context of gender diversity, for example, a trans parent may pass on not only genetic variants influencing gender diversity but also a home environment conducive to the expression of gender diversity, creating a correlation between genes and environment. Taking into account such correlations is important, since rGE can confound estimates of both the genetic and environmental contributions. Many of the included studies acknowledge this as a limitation of their models, and as such, it should be considered when interpreting our findings. Fifth, it is also important to acknowledge that the data presented in this manuscript reflect population-level averages, and do not speak to the personal and complex experiences of gender diversity (and its origins) for any given individual. Finally, on a related note, it is important to emphasise that our findings should not be used to support views of biological determinism nor to promote theories regarding causal roles for specific environmental factors. As noted earlier, the twin studies we reviewed here were not designed to address the role of individual factors, either genetic or environmental.

## Conclusion

This review of the twin literature suggests that there is likely both heritable and non-shared environmental contributions to gender diversity, but the magnitude of these contributions remains undefined and further research is needed to help clarify this.

## Supplementary Information

Below is the link to the electronic supplementary material.

Supplementary Material 1

## Data Availability

No datasets were generated or analysed during the current study.

## References

[CR1] Achenbach TM (1991) Manual for the child behavior checklist/ 4–18 and 1991 profile. University of Vermont Department of Psychiatry, Burlingston

[CR2] Ahrenfeldt LJ, Christensen K, Segal NL, Hur YM (2020) Opposite-sex and same-sex twin studies of physiological cognitive and behavioral traits. Neurosci Biobehav Rev 108(1):322–340. 10.1016/j.neubiorev.2019.11.004 31711815 10.1016/j.neubiorev.2019.11.004 PMC6949417

[CR3] Åhs JW, Dhejne C, Magnusson C, Dal H, Lundin A, Arver S, Dalman C, Kosidou K (2018) Proportion of adults in the general population of Stockholm County who want gender-affirming medical treatment. PLoS One 13(10):e0204606. 10.1371/journal.pone.0204606 30289896 10.1371/journal.pone.0204606 PMC6173394

[CR4] Alanko K, Santtila P, Harlaar N, Witting K, Varjonen M, Jern P, Johansson A, von der Pahlen B, Sandnabba NK (2009) Common genetic effects of gender atypical behavior in childhood and sexual orientation in adulthood: A study of Finnish twins. Arch Sex Behav 39:1–12. 10.1007/s10508-008-9457-3 10.1007/s10508-008-9457-3 19172387

[CR52] American Psychological Association (2015) Guidelines for psychological practice with transgender and gender nonconforming people. Am Psychol 70(9):832–864. 10.1037/a0039906 26653312 10.1037/a0039906

[CR5] Bailey JM, Dunne MP, Martin NG (2000) Genetic and environmental influences on sexual orientation and its correlates in an Australian twin sample. J Personality Social Psychol 78(3):524–536 10.1037//0022-3514.78.3.524 10743878

[CR6] van Beijsterveldt CE, Hudziak JJ, Boomsma DI (2006) Genetic and environmental influences on cross-gender behavior and relation to behavior problems: a study of Dutch twins at ages 7 and 10 years. Arch Sex Behav 35(6):647–658. 10.1007/s10508-006-9072-0 17109235 10.1007/s10508-006-9072-0

[CR7] Buhrich N, Bailey JM, Martin NG (1991) Sexual orientation sexual identity and sex-dimorphic behaviors in male twins. Behav Genet 21:75–96. 10.1007/BF01067668 2018464 10.1007/BF01067668

[CR8] Burri A, Cherkas L, Spector T, Rahman Q (2011) Genetic and environmental influences on female sexual orientation childhood gender typicality and adult gender identity. PLoS One. 10.1371/journal.pone.0021982 21760939 10.1371/journal.pone.0021982 PMC3131304

[CR53] Cass H (2024) The Cass Review. https://cass.independent-review.uk/home/publications/final-report/

[CR9] Cloninger CR, Przybeck TR, Svrakic DM (1991) The tridimensional personality questionnaire: U.S. normative data. Psychol Rep 69(3):1047–1057. 10.2466/pr0.1991.69.3.1047 1784653 10.2466/pr0.1991.69.3.1047

[CR10] Coleman E, Radix AE, Bouman WP et al (2022) Standards of care for the health of transgender and gender diverse people version 8. Int J Transgender Health 23:1–259. 10.1080/26895269.2022.2100644 10.1080/26895269.2022.2100644 PMC9553112 36238954

[CR11] Coolidge FL, Thede LL, Young SE (2002) The heritability of gender identity disorder in a child and adolescent twin sample. Behav Genet 32(4):251–257. 10.1023/a:1019724712983 12211624 10.1023/a:1019724712983

[CR12] Diamond M (2013) Transsexuality among twins: identity concordance transition rearing and orientation. Int J Transgenderism 14(1):24–38. 10.1080/15532739.2013.750222

[CR13] Eysenck HJ, Eysenck SBG (1975) Manual for the Eysenck personality questionnaire. Hodder & Stoughton, London

[CR14] Eysenck SBG, Eysenck HJ, Barrett P (1985) A revised version of the psychoticism scale. Pers Individ Differ 6(1):21–29. 10.1016/0191-8869(85)90026-1

[CR15] Falconer DS (1965) The inheritance of liability to certain diseases estimated from the incidence among relatives. Ann Hum Genet 29(1):51–76. 10.1111/j.1469-1809.1965.tb00500.x

[CR54] Finn SE (1987) The structure of masculinity and femininity self ratings. Unpublished manuscript

[CR16] Foreman M, Hare L, York K, Balakrishnan K, Sánchez FJ, Harte F, Erasmus J, Vilain E, Harley VR (2019) Genetic link between gender dysphoria and sex hormone signaling. J Clin Endocrinol Metab 104(2):390–396. 10.1210/jc.2018-01105 30247609 10.1210/jc.2018-01105

[CR17] Golombok S, Rust J (1993) The measurement of gender role behaviour in pre-school children: a research note. J Child Psychol Psychiatry; Allied Disciplines 34(5):805–811. 10.1111/j.1469-7610.1993.tb01072.x 10.1111/j.1469-7610.1993.tb01072.x 8340446

[CR18] Hall JA, Halberstadt AG (1980) Masculinity and femininity in children: development of the children’s personal attributes questionnaire. Dev Psychol 16(4):270–280. 10.1037/0012-1649.16.4.270

[CR19] Hayden JA, Côté P, Bombardier C (2006) Evaluation of the quality of prognosis studies in systematic reviews. Ann Intern Med 144(6):427–437. 10.7326/0003-4819-144-6-200603210-00010 16549855 10.7326/0003-4819-144-6-200603210-00010

[CR20] Hayden JA, van der Windt DA, Cartwright JL et al (2013) Assessing bias in studies of prognostic factors. Ann Intern Med 158(4):280–286. 10.7326/0003-4819-158-4-201302190-00009 23420236 10.7326/0003-4819-158-4-201302190-00009

[CR21] Heylens G, De Cuypere G, Zucker KJ, Schelfaut C, Elaut E, Vanden Bossche H, De Baere E, T’Sjoen G (2012) Gender identity disorder in twins: A review of the case report literature. J Sex Med 9(3):751–757. 10.1111/j.1743-6109.2011.02567.x 10.1111/j.1743-6109.2011.02567.x 22146048

[CR22] Hines M (2011a) Gender development and the human brain. Annu Rev Neurosci 34(1):69–88. 10.1146/annurev-neuro-061010-113654 21438685 10.1146/annurev-neuro-061010-113654

[CR23] Hines M (2011b) Prenatal endocrine influences on sexual orientation and on sexually differentiated childhood behavior. Front Neuroendocrinol 32(2):170–182. 10.1016/j.yfrne.2011.02.006 21333673 10.1016/j.yfrne.2011.02.006 PMC3296090

[CR24] Iervolino AC, Hines M, Golombok SE, Rust J, Plomin R (2005) Genetic and environmental influences on sex-typed behavior during the preschool years. Child Dev 76(4):826–840. 10.1111/j.1467-8624.2005.00880.x 16026499 10.1111/j.1467-8624.2005.00880.x

[CR25] John OP, Donahue EM, Kentle RL (1991) The big five Inventory – Versions 4a and 5. University of California Berkeley Institute of Personality and Social Research, Berkeley California

[CR26] Karamanis G, Karalexi M, White R, Frisell T, Isaksson J, Skalkidou A, Papadopoulos FC (2022) Gender dysphoria in twins: a register-based population study. Sci Rep 12:13439. 10.1038/s41598-022-17749-0 35927439 10.1038/s41598-022-17749-0 PMC9352732

[CR27] Knafo A, Iervolino AC, Plomin R (2005) Masculine girls and feminine boys: genetic and environmental contributions to atypical gender development in early childhood. J Pers Soc Psychol 88(2):400–412. 10.1037/0022-3514.88.2.400 15841866 10.1037/0022-3514.88.2.400

[CR28] Lippa R, Hershberger S (1999) Genetic and environmental influences on individual differences in masculinity femininity and gender diagnosticity: analyzing data from a classic twin study. J Pers 67(1):127–155. 10.1111/1467-6494.00050 10030022 10.1111/1467-6494.00050

[CR29] Loehlin JC, Jonsson EG, Gustavsson JP, Stallings MC, Gillespie NA, Wright MJ, Martin NG (2005) Psychological masculinity-femininity via the gender diagnosticity approach: heritability and consistency across ages and populations. J Pers 73(5):1295–1320. 10.1111/j.1467-6494.2005.00350.x 16138874 10.1111/j.1467-6494.2005.00350.x

[CR31] McConaghy N, Armstrong MS, Birrell PC, Buhrich N (1979) The incidence of bisexual feelings and opposite sex behavior in medical students. J Nerv Ment Dis 167(11):685–688. 10.1097/00005053-197911000-00005 501343 10.1097/00005053-197911000-00005

[CR32] Meyer-Bahlburg HFL, Dolezal C, Baker SW, Ehrhardt AA, New MI (2006) Gender development in women with congenital adrenal hyperplasia as a function of disorder severity. Arch Sex Behav 35(6):667–684. 10.1007/s10508-006-9068-9 16902816 10.1007/s10508-006-9068-9

[CR33] Miller EM (1994) Prenatal sex hormone transfer: a reason to study opposite-sex twins. Pers Individ Differ 17(4):511–529. 10.1016/0191-8869(94)90088-4

[CR34] Mitchell JE, Baker LA, Jacklin CN (1989) Masculinity and femininity in twin children: genetic and environmental factors. Child Dev 60(6):1475–1485. 10.2307/1130936 2612253

[CR35] Neale MC, Cardon LR (1992) Methodology for genetic studies of twins and families. 10.1007/978-94-015-8018-2. Kluwer Academic/Plenum Publishers

[CR46] Page MJ, McKenzie JE, Bossuyt PM, Boutron I, Hoffmann TC, Mulrow CD et al (2021) The PRISMA 2020 statement: an updated guideline for reporting systematic reviews. 10.1136/bmj.n71. BMJ 372 n71 10.1136/bmj.n71 PMC8005924 33782057

[CR36] Pasterski V, Zucker KJ, Hindmarsh PC, Hughes IA, Acerini C, Spencer D, Neufeld S, Hines M (2015) Increased cross-gender identification independent of gender role behavior in girls with congenital adrenal hyperplasia: results from a standardized assessment of 4- to 11-year-old children. Arch Sex Behav 44(5):1363–1375. 10.1007/s10508-014-0385-0 25239661 10.1007/s10508-014-0385-0

[CR37] Plomin R, DeFries JC, Loehlin JC (1977) Genotype-environment interaction and correlation in the analysis of human behaviour. Psychol Bull 84(2):309–322. 10.1037/0033-2909.84.2.309 557211

[CR38] Polderman TJC, Benyamin B, de Leeuw CA, Sullivan PF, van Bochoven A, Visscher PM, Posthuma D (2015) Meta-analysis of the heritability of human traits based on fifty years of twin studies. Nat Genet 47(7):702–709. 10.1038/ng.3285 25985137 10.1038/ng.3285

[CR39] Polderman TJC, Kreukels BPC, Irwig MS, Beach L, Chan YM, Derks EM, Esteva I, Ehrenfeld J, Heijer MD, Posthuma D, Raynor L, Tishelman A, Davis LK (2018) The biological contributions to gender identity and gender diversity: bringing data to the table. Behav Genet 48(2):95–108. 10.1007/s10519-018-9889-z 29460079 10.1007/s10519-018-9889-z

[CR40] Ristori J, Cocchetti C, Romani A, Mazzoli F, Vignozzi L, Maggi M, Fisher AD (2020) Brain sex differences related to gender identity development: genes or hormones?? Int J Mol Sci 21(6):2123. 10.3390/ijms21062123 32204531 10.3390/ijms21062123 PMC7139786

[CR41] Ronald A, de Bode N, Polderman TJC (2021) How the attentiondeficit/hyperactivity disorder polygenic risk score adds to our Understanding of ADHD and associated traits: A systematic review. J Am Acad Child Adolesc Psychiatry 60(10):1234–1277. 10.1016/j.jaac.2021.01.019 33548493 10.1016/j.jaac.2021.01.019 PMC11164195

[CR42] Sasaki S, Ozaki K, Yamagata S, Takahashi Y, Shikishima C, Kornacki T, Nonaka K, Ando J (2016) Genetic and environmental influences on traits of gender identity disorder: a study of Japanese twins across developmental stages. Arch Sex Behav 45(7):1681–1695. 10.1007/s10508-016-0821-4 27507021 10.1007/s10508-016-0821-4

[CR43] Schalling D, Asberg M, Edman G, Oreland L (1987) Markers for vulnerability to psychopathology: temperament traits associated with platelet MAO activity. Acta Psychiatr Scand 76(2):172–182. 10.1111/j.1600-0447.1987.tb02881.x 3673640 10.1111/j.1600-0447.1987.tb02881.x

[CR55] Statistics Sweden (2024) ‘Population by sex and country of birth 1970–2023 and projection 2024–2070 [Data set]. https://www.scb.se/en/finding-statistics/statistics-by-subject-area/population/population-projections/population-projections/pong/tables-and-graphs/population-by-sex--age-and-country-of-birth-and-projection/

[CR44] Steensma TD, McGuire JK, Kreukels BP, Beekman AJ, Cohen-Kettenis PT (2013) Factors associated with desistence and persistence of childhood gender dysphoria: a quantitative follow-up study. J Am Acad Child Adolesc Psychiatry 52(6):582–590. 10.1016/j.jaac.2013.03.016 23702447 10.1016/j.jaac.2013.03.016

[CR45] Tancredy CM, Fraley RC (2006) The nature of adult twin relationships: an attachment-theoretical perspective. J Personality Soc Psychol 90(1):78–93. 10.1037/0022-3514.90.1.78 10.1037/0022-3514.90.1.78 16448311

[CR47] Theisen JG, Sundaram V, Filchak MS, Chorich LP, Sullivan ME, Knight J, Kim HG, Layman LC (2019) The use of whole exome sequencing in a cohort of transgender individuals to identify rare genetic variants. Sci Rep 9:20099. 10.1038/s41598-019-53500-y 31882810 10.1038/s41598-019-53500-y PMC6934803

[CR48] Thomas SJ (1983) Providing a concrete context for sex role endorsement in adolescents: Some measurement considerations [Paper presentation]. Annual meeting of the American Educational Research Association Montreal

[CR49] Twist J, de Graaf NM (2019) Gender diversity and non-binary presentations in young people attending the United Kingdom’s National gender identity development service. Clin Child Psychol Psychiatry 24(2):277–290. 10.1177/1359104518804311 30326742 10.1177/1359104518804311

[CR50] Verweij KJ, Mosing MA, Ullen F, Madison G (2016) Individual differences in personality masculinity-femininity: examining the effects of genes, environment and prenatal hormone transfer. Twin Res Hum Genet 19(2):87–96. 10.1017/thg.2016.8 26948461 10.1017/thg.2016.8

[CR51] Zucker KJ, Mitchell JN, Bradley SJ, Tkachuk J, Cantor JM, Allin S (2006) The recalled childhood gender identity/gender role questionnaire: psychometric properties. Sex Roles 54:469–483. 10.1007/s11199-006-9019-x

