# Supplementary material for: Genetic and Environmental Contributions To Gender Diversity: A Systematic Review of the Twin Literature
Source: Behav Genet. 2025 Sep 12;55(5):339–59. doi: 10.1007/s10519-025-10231-3 (PMC12494644; doi:10.1007/s10519-025-10231-3)
Supplement: Supplementary file 1 — Supplementary Material 1 [file 10519_2025_10231_MOESM1_ESM.docx]

**SUPPLEMENTARY MATERIALS**

**1. Medline Search Terms**

(Transgender Persons/ or Transsexualism/ or bisexuality/ or homosexuality/ or Gender Dysphoria/ or sex reassignment procedures/ or sex reassignment surgery/ or Gender Identity/ or (gender-identity or gender-dysphori* or gender-varian* or genderqueer* or queer* or intersex* or inter-sex* or transgender* or trans-gender* or transsexual* or trans-sexual or transexual* or two-spirit or trans-m#n or transm#n or trans-male* or transmale* or trans-wom#n or transwom#n or trans-female* or transfemale* or transfeminine or trans-feminine or transmasculine or trans-masculine or trans-folk* or transfolk* or trans-folx* or transfolx* or trans-people or transpeople or trans-person* or transperson* or trans-spectrum or AFAB or AMAB or cross-gender or QTPOC or M2F or MTF or F2M or FTM or gender-minorit* or sexual-minorit* or non-binary or nonbinary or gender-fluid* or genderfluid* or agender or gender-non-conform* or gender-nonconform* or TGNC or gender-ambigu* or gender-expression or gender-expansive or gender-diverse or transvesti* or third-gender or bisexual* or bi-sexual* or sexual-dissident* or gay or gays or homosexual* or homo-sexual* or GLBT* or LGB or LGBs or LGBT* or lesbian* or lesbigay* or lesbi-gay* or men-who-have-sex-with-men or non-heterosexual* or nonheterosexual* or women-who-have-sex-with-women or gender-transition* or gender-creative or Gender-change or sex-reassignment or gender-reassignment or gender-confirmation or gender-affirmation or sex-change or sex-transformation or gender-transformation).tw,kf.)

AND

(Genetics/ or phenotype/ or gene-environment interaction/ or genetic markers/ or Genotype/ or genome/ or epigenome/ or exome/ or genome, human/ or ge.fs. or (heritab* or genetic* or inherit* or predispos* or concord* or aetiolog* or etiolog* or gene-environment).tw,kf.)

AND

(Multiple Birth Offspring/ or (twin or twins or twinning or monozygot* or dizygo* or triplet* or quadruplet* or quintuplet*).tw,kf.)

**2. Embase Search Terms**

(Transgender/ or LGBT people/ or “transgender and gender nonbinary”/ or female to male transgender/ or male to female transgender/ or gender dysphoria/ or sex reassignment/ or transsexualism/ or gender identity/ or [(gender-identity or gender-dysphori* or gender-varian* or genderqueer* or queer or queers or intersex* or inter-sex* or transgender* or trans-gender* or transsexual* or trans-sexual or transexual* or two-spirit or trans-m#n or transm#n or trans-male* or transmale* or trans-wom#n or transwom#n or trans-female* or transfemale* or transfeminine or trans-feminine or transmasculine or trans-masculine or trans-folk* or transfolk* or trans-folx* or transfolx* or trans-people or transpeople or trans-person* or transperson* or trans-spectrum or afab or amab or cross-gender or qtpoc or m2f or mtf or f2m or ftm or gender-minorit* or sexual-minorit* or non-binary or nonbinary or gender-fluid* or genderfluid* or agender or gender-non-conform* or gender-nonconform* or tgnc or gender-ambigu* or gender-expression or gender-expansive or gender-diverse or transvesti* or third-gender or bisexual* or bi-sexual* or sexual-dissident* or gay or gays or homosexual* or homo-sexual* or glbt* or lgb or lgbs or lgbt* or lesbian* or lesbigay* or lesbi-gay* or men-who-have-sex-with-men or non-heterosexual* or nonheterosexual* or women-who-have-sex-with-women or gender-transition* or gender-creative or gender-change or sex-reassignment or gender-reassignment or gender-confirmation or gender-affirmation or sex-change or sex-transformation or gender-transformation).tw,hw,dq,kf.])

AND

(Etiology/ or Genetics/ or Phenotype/ or Genotype Phenotype Interaction/ or Genetic Marker/ or Epigenome/ or Genome/ or Environmental Factor/ or [(heritab* or genetic* or inherit* or predispos* or concord*).tw,hw,dq,kf.])

AND

(Twins/ or triplets/ or multiple birth offspring/ or quadruplets/ or quintuplets/ or [(twin or twins or triplet* or quadruplet* or quintuplet*).tw,hw,dq,kf.])

**3. PubMed Search Terms**

(gender-identity OR gender-dysphori* OR gender-varian* OR genderqueer* OR queer OR queers OR intersex* OR inter-sex* OR transgender* OR trans-gender* OR transsexual* OR trans-sexual OR transexual* OR two-spirit OR trans-m#n OR transm#n OR trans-male* OR transmale* OR trans-wom#n OR transwom#n OR trans-female* OR transfemale* OR transfeminine OR trans-feminine OR transmasculine OR trans-masculine OR trans-folk* OR transfolk* OR trans-folx* OR transfolx* OR trans-people OR transpeople OR trans-person* OR transperson* OR trans-spectrum OR AFAB OR AMAB OR cross-gender OR QTPOC OR M2F OR MTF OR F2M OR FTM OR gender-minorit* OR sexual-minorit* OR non-binary OR nonbinary OR gender-fluid* OR genderfluid* OR agender OR gender-non-conform* OR gender-nonconform* OR TGNC OR gender-ambigu* OR gender-expression OR gender-expansive OR gender-diverse OR transvesti* OR third-gender OR bisexual* OR bi-sexual* OR sexual-dissident* OR gay OR gays OR homosexual* OR homo-sexual* OR GLBT* OR LGB OR LGBs OR LGBT* OR lesbian* OR lesbigay* OR lesbi-gay* OR men-who-have-sex-with-men OR non-heterosexual* OR nonheterosexual* OR women-who-have-sex-with-women OR gender-transition* OR gender-creative OR Gender-change OR sex-reassignment OR gender-reassignment OR gender-confirmation OR gender-affirmation OR sex-change OR sex-transformation OR gender-transformation)

AND

(Genetic OR genetics OR allele OR alleles OR allelic OR homozygous OR heterozygous OR homozygote OR heterozygote OR epigenetics OR epigenome OR epigenomics OR exome OR exomes OR gene OR genes OR genome OR genomes OR GWAS OR genotype OR genotypes OR phenotype OR phenotypes OR haploid OR haploids OR haplotype OR haplotypes OR genome-wide-analysis OR hemizygous OR homozygosity OR heterozygosity OR polygenic OR monogenic OR penetrance OR pleiotropy OR polymorph* OR variant OR heritable OR heritability OR inheritance OR inherited OR inherit* OR herit* OR concordant OR concordance OR etiology OR aetiology OR environment OR gene-environment OR gene-environment-interaction OR environmental)

AND

(Twin OR Twins OR Triplet OR Triplets OR Monozygot* OR Dyzygot* OR Quadruplet* OR Quituplet* OR Multiple Birth Offspring)
